# Daith Piercing in a Case of Chronic Migraine: A Possible Vagal Modulation

**DOI:** 10.3389/fneur.2017.00624

**Published:** 2017-11-27

**Authors:** Angelo Cascio Rizzo, Matteo Paolucci, Riccardo Altavilla, Nicoletta Brunelli, Federica Assenza, Claudia Altamura, Fabrizio Vernieri

**Affiliations:** ^1^Headache and Neurosonology Unit, Neurology, Policlinico Campus Bio-Medico di Roma, Rome, Italy; ^2^Stroke Unit, Medicina vascolare e d’urgenza, Università di Perugia, Perugia, Italy

**Keywords:** ear acupuncture, medication overuse headache, vagus nerve, neuromodulation, transcutaneous vagal stimulation

## Abstract

Daith piercing is an ear piercing located at the crus of the helix, bilaterally. It is getting great consent on social media as alternative treatment in chronic migraine. No data about its efficacy and action are available in scientific literature so far. We present the case of a 54-year-old male patient suffering from refractory chronic migraine with medication-overuse, who substantially improved after bilateral ear daith piercing. His migraine was refractory to symptomatic as well as prophylactic therapies. He used to treat headaches with up to five symptomatic drugs per attack and had attempted several pharmacological preventive therapies, including Onabotulinumtoxin A. He also underwent detoxification treatments with intravenous steroids and diazepam, without durable benefit. At the time of daith piercing, the headache-related disability measures showed a HIT-6 score of 64, a MIDAS-score of 70, and a 11-point Box scale of 5. On his own free will, he decided to get a “daith piercing.” After that, he experienced a reduction of migraine attacks, which became very rare, and infrequent, less disabling episodes of tension-type headache (HIT-6 score of 56; MIDAS score of 27, 11-point Box scale of 3). Painkiller assumption has much decreased: he takes only one tablet of indomethacin 50 mg to treat tensive headaches, about four times per month. Beyond a placebo effect, we can speculate a vagal modulation as the action mechanism of daith piercing: a nociceptive sensory stimulus applied to trigeminal and vagal areas of the ear can activate ear vagal afferents, which can modulate pain pathways by means of projections to the caudal trigeminal nucleus, to the locus coeruleus and to the nucleus raphe magnus. Currently, daith piercing cannot be recommended as migraine treatment because of the lack of scientific evidence, the unquantified rate of failure and the associated risks with insertion. However, given the increasing but anecdotal evidence, we think that the mechanism needs testing by means of a controlled clinical trial in a population of chronic migraineurs.

## Introduction

**Figure 1:**
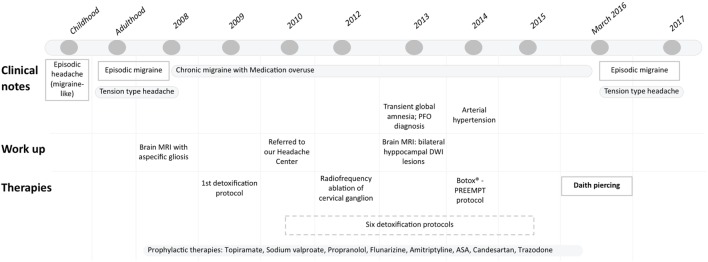
Timeline of patient’s clinical history from childhood to daith piercing insertion.

**Figure 2:**
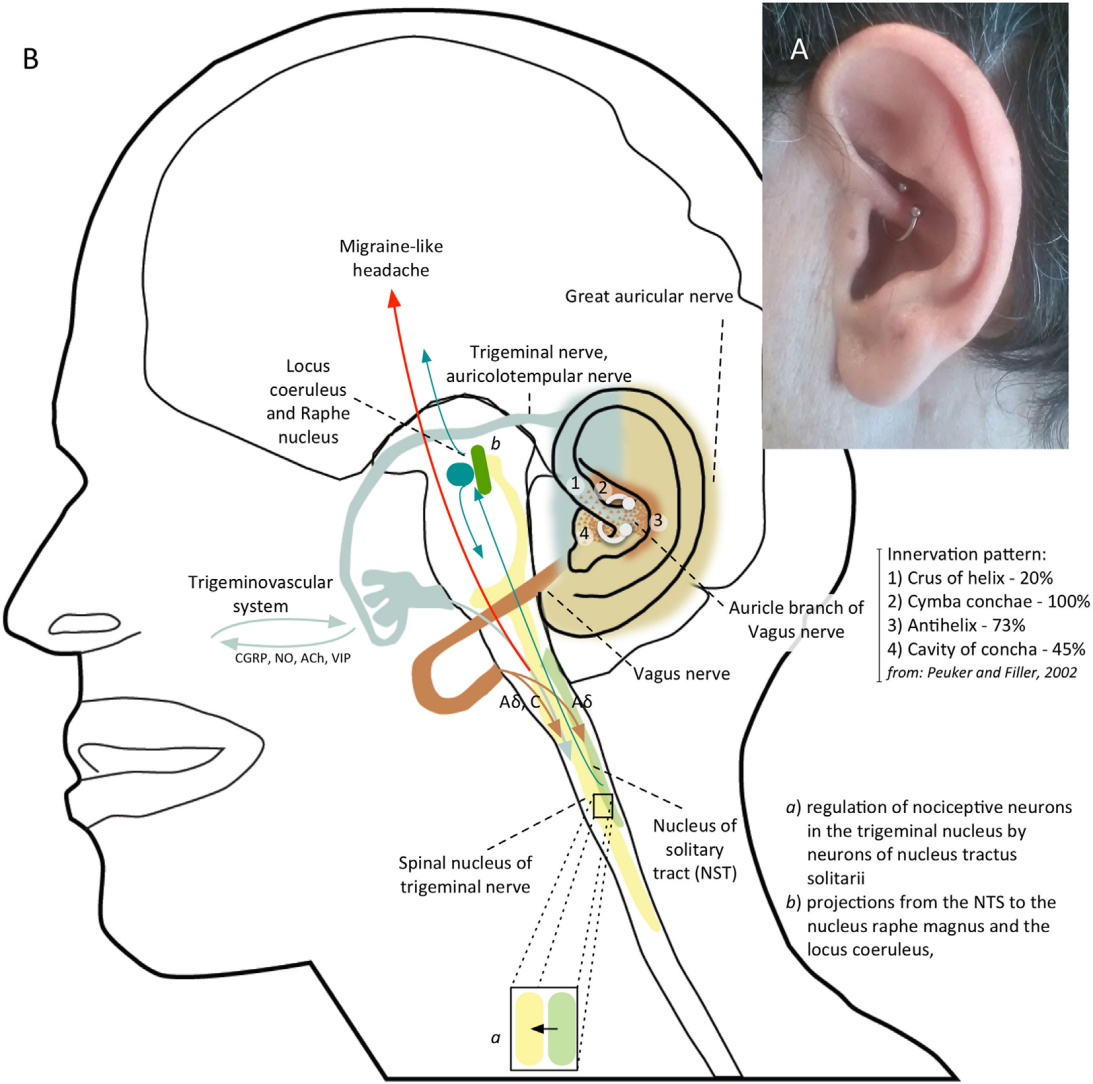
**(A)** Patient’s left ear with daith piercing located at the crus of the helix. **(B)** Shows the possible mechanism of action of daith piercing: a nociceptive sensory stimulus provided by daith piercing activates vagal afferents which, through the nucleus tractus solitarii (NTS), exert an inhibitory action on neurons in the caudal trigeminal nucleus; vagal activation can also modulate pain perception by means projections from NTS to the locus coeruleus and to the nucleus raphe magnus.

We present the case of a 54-year-old male patient, suffering from headache since childhood. His family history is positive for migraine and his past medical history includes a head trauma occurred when he was 3 years old. During the years, the patient has presented two types of headache with variable frequency: migraine without aura since school-age, and a disabling tension-type headache, started in adulthood. Sleep deprivation, alcohol, traveling represent his main trigger factors. His clinical history is summarized in Figure 1. Since 2008, his migraine became “chronic” according to ICHD-3beta version definition (1). At that time, neurological examination was normal and brain MRI showed two subcortical small high T2-weighted lesions, diagnosed as aspecific gliosis. To counteract this chronic headache, he has been prescribed several preventive therapies including topiramate, sodium valproate, propranolol, flunarizine, amitriptyline at the recommended therapeutic dosage according to guidelines (2). In most cases, the patient reported a transient benefit on headache frequency and intensity, lasting only few months. He treated acute episodes with multiple painkillers, i.e., triptans and nonsteroidal anti-inflammatory drugs (NSAIDs), up to five symptomatic drugs per attack. Hence, he was also diagnosed a medication overuse headache (MOH). In 2009, the patient underwent a first detoxification treatment with glutathione, cyanocobalamin, folic acid, nicotinamide, ascorbic acid, delorazepam, and metoclopramide, without any benefit. In 2010, our headache center started taking care of the patient. At that time, he suffered from chronic migraine and MOH. The most effective symptomatic drug was indomethacin 50 mg, which he took almost on a daily basis. He underwent a second detoxification treatment with intravenous steroids (prednisone) and diazepam at our hospital. The treatment was effective only for few weeks, then migraine returned chronic and the patient resumed to abuse symptomatic drugs. Moreover, since he had to travel abroad very often, he started experiencing anticipatory fear of suffering from severe headaches, making painkiller and steroid assumption more frequent. Then, the patient received six detoxification treatments with intravenous steroids and diazepam, almost twice per year, until 2015. These treatments were necessary as they provided relief and allowed the patient to attend his work. After each detoxification treatment, a short cycle of oral methylprednisolone and different preventive therapies (i.e., topiramate, amitriptyline, trazodone, propranolol) were prescribed. After a transient improvement, headache returned daily once again. In 2012, he underwent radiofrequency ablation of cervical ganglion without clinical benefit. In 2013, the patient presented a transient global amnesia, brain MRI evidenced an acute hippocampal lesion, transcranial doppler, and echocardiography with bubble test showed a patent foramen ovale with atrial septal aneurysm. We prescribed aspirin 100 mg/day. In 2014, arterial hypertension was diagnosed, so he started taking candesartan 16 mg/day for his additional action in preventing migraine. Given the persistence of headache and failure of the preventive drugs, Onabotulinumtoxin-A treatment was started in September 2014 for 1 year, according to PREEMPT protocol. He still presented 12–13 attacks per month, but a reduction of the headache intensity and, to a lesser extent, of painkillers assumption (i.e., zolmitriptan, indomethacin, paracetamol) was observed. At that time headache-related disability measures showed a HIT-6 score of 64, a MIDAS-score of 70, a headache intensity of 5 out 10 at 11-point Box scale (BS-11). Since the repeated therapeutic attempts led only to minimum benefit, in March 2016, the patient decided, on his own free will, to get a “daith piercing,” which is an ear piercing located at the crus of the helix (Figure 2A), bilaterally. At that time, he was on therapy with amitriptyline 25 mg/bid, aspirin 100 mg/day, he suffered from headache at least 15 days per month and took up to 15 painkillers per month. On the following months, he experienced an important reduction of migraine attacks, which returned episodic, and infrequent, less disabling episodes of tension-type headache. Head pain has become less intense than before, with a score of 3 out 10 at BS-11: he describes pain as oppressive and annoying, affecting concentration but rarely interfering with his work and other daily activities (HIT-6 score 56). In addition, he has suffered from headache only 13 days in the last 3 months (MIDAS-score 27) and treated each with a single painkiller. Today, after one and half year, the patient is satisfied and can better attend to his work, traveling and alcohol do not trigger headaches anymore. Migraine attacks are very rare (none in the last 2 months) and he only suffers from infrequent episodic tensive-type headache. He currently takes amitriptyline 25 mg per day. Painkiller assumption is much decreased: he takes only one tablet of indomethacin 50 mg to treat attacks, about four times per month.

## Background

Preventive therapy for chronic migraine is used to reduce the frequency, duration, or severity of attacks. Several treatments can alleviate headache, but their effect may fail, or be partial and inconstant across individuals. For this reason, potential alternative migraine treatments, without definitive scientific evidence, are getting great consent on social media. A growing number of migraine sufferers has experimented the use of a particular alternative treatment known as “daith piercing” that is a piercing located in the ear cartilage at the crus of helix.

## Discussion

No data on daith piercing are available in scientific literature. An anonymous survey promoted online (https://blog.migrainepal.com) investigated its effect on attack frequency and intensity. Of the 380 patients with a daith piercing completing the survey, 47.2% experienced a reduction in migraine frequency. Of the remainder half, 4.7% got worse and the others did not show any change. Half (49.9%) of the responders experienced less severe attacks. However, the number of responders who had no further migraine attacks decreased over the months. It is not intuitive how a piercing can provide a clinical benefit in migraine. Interestingly, daith piercing involves similar ear areas of auricular acupuncture, suggesting a similar action mechanism. Prior studies demonstrated that acupuncture can be helpful in the treatment of migraine (3) with acute and long-term benefit compared with sham acupuncture (4). Different studies conducted using auricular acupuncture showed a benefit on migraine pain control within 30 min and up to 24 h from the semi-permanent needle insertion in specific auricular points in adults (5, 6). Moreover, treating specific auricular points seems to be more effective in controlling migraineurs pain (7). A study comparing somatic versus ear acupuncture for migraine without aura treatment showed promising results regarding ear acupuncture efficacy (8). A systematic review and meta-analysis, including a sham control group, suggested that auricular therapy can be used as an adjunct therapy for pain management (9). The therapeutic mechanism of ear acupuncture is not well known. Some theories suggest that acupuncture stimulates sensory nerves of the skin and body muscles, causing significant release of β-endorphin, or acting as a non-painful sensory stimulus that competitively inhibits nociceptive pathways according to the classical gate-control model by Melzack and Wall. The auricular branch of the vagus nerve, the auriculotemporal branch of the trigeminal nerve, the great auricular nerve from the second and third cervical roots as well as the facial and glossopharyngeal nerve concur to somatic innervation of the ear surface. The auricular branches of the trigeminal and vagus nerve are responsible for 80 and 20% of sensory innervation of anterior part of the helix, respectively (10). The sensory stimulus provided by a needle or piercing insertion in this site can modulate the trigeminovascular system, that is the pathway behind migraine headaches, acting on the nociceptive input. Several fMRI studies showed how acupuncture can affect structures modulating trigeminal nociceptive input including the rostral ventromedial medulla, ventrolateral periaqueductal gray, locus coeruleus, and the nucleus raphe magnus (11, 12). Moreover, acupuncture determines a change in connectivity in pain [anterior cingulate cortex (ACC), periaqueductal gray], affective (amygdala, ACC), and memory (hippocampus, middle temporal gyrus) related brain regions (13). The stimulation of specific points of the ear by acupuncture produces an fMRI activation of the pain matrix areas (somatosensory and limbic areas) involved in the processing of the affective-cognitive components of pain perception (14). Functional MRI studies using electrical stimulation (versus sham) of vagal ear areas evidenced a BOLD signal decreasing in the area of the nuclei of the vagus nerve in the brainstem and in the area of the pain matrix reached by vagal projections, indicating an effective stimulation of vagal afferences (15–17). Given the contribution of vagus nerve in the innervation of the helix, a vagal activation may be responsible of the benefit of acupuncture (18) and daith piercing. The action mechanism is probably multifactorial. Vagal stimulation can exerts a possible inhibitory action on nociceptive neurons in the caudal trigeminal nucleus through possible reciprocal connections between the caudal trigeminal nucleus and the nucleus tractus solitarii (NTS), which is the major target of vagal afferents (19) (Figure 2B). Vagal stimulation can also modify cortical excitability, which is altered in chronic migraine, through projections from NTS to the locus coeruleus, to the nucleus raphe magnus and to several subcortical and cortical regions, including thalamus, insula, and lateral prefrontal cortex, which are involved in the pain matrix (19). The anti-nociceptive effect could be also due to the activation of these descending inhibitory pathways.

Stimulation of NTS through auricular vagal projections could also have an autonomic effect, reducing sympathetic output through projections in the medulla, and increasing parasympathetic output activating the dorsal motor nucleus of the vagus and the nucleus ambiguous (20), thus modifying cortical excitability. Alternatively, a modulation of the release from vagal efferents on dural vessels, of neurotransmitters and inflammatory molecules (21) involved in neurogenic inflammation and sensitization can be hypothesized. Approaching vagal nerve by its auricular branch is probably one of the best ways to modulate its effect on the activation of trigeminovascular pathway (19). In fact, the transcutaneous stimulation of the auricular vagal nerve (t-VNS) resulted effective in the treatment of chronic migraine (22). Moreover, a recent study demonstrates a similar preventive action on acute and chronic pain by stimulating ear vagal areas with t-VNS or acupuncture (18). Also, supraorbital trigeminal nerve stimulation has shown a migraine preventive action in a controlled study (23). Our opinion is that a modulation of trigeminovascular pathway through a stimulus applied to trigeminal and vagal areas of the ear can be responsible of the beneficial effect of daith piercing in migraine patients. We do not know how daith piercing, once it has healed, can provide a continuous stimulation of the vagal and trigeminal pathways. The modulation of pain perception induced by piercing may translate in a change of functional connectivity in cerebral areas taking part of the pain matrix that can explain the potential therapeutic effects of daith piercing and of ear acupuncture.

## Concluding Remarks

There have been growing amounts of positive as well as negative reports regarding daith piercing on the internet and social media by patients suffering from headache.

We describe the case of one patient with chronic migraine who decided, on his own free will, to get a “daith piercing.” During the last months, we recorded an improvement of migraine attacks but not of tensive-type episodes, supporting the hypothesis that piercing may be specific for the former type of headache. We are aware that the effect on our patient, as well as other anecdotal reports on daith piercing, can be influenced by the placebo effect. Moreover, there are many reports of persisting pain, worsening attacks or slow healing over months. In addition, piercing insertion at this site is associated with a considerable risk for infection. Therefore, although daith piercing may look like an attractive therapeutic option, at the moment, it cannot be recommended for migraine treatment, because of lack of scientific evidence, as well as the unquantified rate of failure and associated risks. The proposed therapeutic mechanism needs testing by means of controlled clinical trials in patients suffering from chronic migraine. In particular, it could help demonstrating an actual role of piercing on disabling migraines that are refractory to consolidated symptomatic and preventive treatments.

## Ethics Statement

Ethics approval and consent was obtained by “Ethics Committee of Campus Bio-Medico University.” Written informed consent was obtained from the patient for publication of this case report and accompanying images.

## Author Contributions

AR and FV: conception of the work; acquisition and interpretation of data; drafting manuscript; critical revision. MP: conception of the work; interpretation of data; manuscript drafting; critical revision. RA, NB, and FA: critical revision. CA: conception of the work; manuscript drafting; critical revision. All authors read and approved the final manuscript.

## Conflict of Interest Statement

The authors declare that the research was conducted in the absence of any commercial or financial relationships that could be construed as a potential conflict of interest. The reviewer JE and handling editor declared their shared affiliation.

## Abbreviations

NSAIDs, nonsteroidal anti-inflammatory drugs; MOH, medication overuse headache; BS-11, 11-point Box scale; ACC, anterior cingulate cortex; NTS, nucleus tractus solitarii; t-VNS, transcutaneous vagal nerve stimulation.
